# Helminth alleviates COVID-19-related cytokine storm in an IL-9-dependent way

**DOI:** 10.1128/mbio.00905-24

**Published:** 2024-05-10

**Authors:** Zengguo Cao, Jiaqi Wang, Xiaolei Liu, Yang Liu, Fangxu Li, Mingyuan Liu, Sandra Chiu, Xuemin Jin

**Affiliations:** 1 State Key Laboratory for Diagnosis and Treatment of Severe Zoonotic Infectious Diseases, Key Laboratory of Zoonosis Research, Ministry of Education, Institute of Zoonosis, College of Veterinary Medicine, Jilin University, Changchun, China; 2 State Key Laboratory of Virology, Key Laboratory of Special Pathogens and Biosafety, Wuhan Institute of Virology, Chinese Academy of Sciences, Wuhan, China; 3 Division of Life Sciences and Medicine, University of Science and Technology of China, Hefei, Anhui, China; University of Calgary, Calgary, Canada

**Keywords:** *Trichinella spiralis*, SARS-CoV-2, cytokine shock, recombinant IL-9

## Abstract

Hyperactivation of pro-inflammatory type 1 cytokines (e.g., tumor necrosis factor alpha [TNF-α] and interferon gamma [IFN-γ]) mirrors the inflammation of coronavirus disease 2019. Helminths could alleviate excessive immune responses. Here, helminth *Trichinella spiralis* (Ts) infection was shown to protect against TNF-α- and IFN-γ-induced shock. Mechanistically, Ts-induced protection was interleukin-9 (IL-9) dependent but not IL-4Rα. Recombinant IL-9 treatment not only improved the survival of wild-type mice with TNF-α- and IFN-γ-induced shock but also that of severe acute respiratory syndrome coronavirus 2 (SARS-CoV-2)-infected K18-human angiotensin-converting enzyme 2 (hACE2) mice, emphasizing the significance of IL-9 in alleviating cytokine storm syndromes during SARS-CoV-2 infection. Interestingly, Ts excretory/secretory (TsES)-induced protection was also observed in SARS-CoV-2 infection, indicating that identifying anti-inflammatory molecules from TsES could be a novel way to mitigate adverse pathological inflammation during pathogen infection.

**IMPORTANCE:**

Severe coronavirus disease 2019 (COVID-19) is linked to cytokine storm triggered by type 1 pro-inflammatory immune responses. TNF-α and IFN-γ shock mirrors cytokine storm syndromes, including COVID-19. Helminths (e.g., *Trichinella spiralis*, Ts) can potently activate anti-inflammatory type 2 immune response. Here, we found that helminth Ts-induced protection against TNF-α and IFN-γ shock was IL-9 dependent. Treatment with recombinant IL-9 could protect against severe acute respiratory syndrome coronavirus 2 (SARS-CoV-2) infection in K18-hACE2 mice. Helminth Ts excretory/secretory (TsES) products also ameliorated SARS-CoV-2 infection-related cytokine storm. In conclusion, our study emphasizes the significance of IL-9 in protecting from cytokine storm syndromes associated with SARS-CoV-2 infection. Anti-inflammatory molecules from TsES could be a new source to mitigate adverse pathological inflammation associated with infections, including COVID-19.

## INTRODUCTION

Helminth infections are often asymptomatic, so-called “old friends,” suggesting a long evolutionary coadaptation between these helminths and humans for hundreds of millions of years. The hygiene hypothesis suggests that the increase in sanitation in developed countries has led to a rise in inflammatory disorders, which may be connected to a decrease in helminth infections. Helminth infections may reduce pro-inflammatory cytokines and immune responses (1). Cytokine storm represents a significant cause of morbidity and mortality in patients with coronavirus disease 2019 (COVID-19) (2). To date, the COVID-19 pandemic has accounted for nearly 7 million deaths globally, which is caused by severe acute respiratory syndrome coronavirus 2 (SARS-CoV-2) (3–5). However, therapeutics that are effective against cytokine storm to treat lethal COVID-19 are still urgently needed.

Viruses typically trigger a type 1 immune response (6, 7). Hyperactivation of pro-inflammatory type 1 cytokines (e.g., tumor necrosis factor alpha [TNF-α] and interferon gamma [IFN-γ]) cause a lethal cytokine shock in the host that mirrors the tissue damage and inflammation of COVID-19 (8). While the onset of these inflammatory responses is vital for managing deadly viral infections, the downside is the potential for harmful tissue inflammation (9). Research conducted in Africa indicates that fewer individuals who contract SARS-CoV-2 experience severe cases of COVID-19 compared to those in developed countries. Coinfection with helminths seems to decrease the likelihood of severe COVID-19 symptoms (10). Recent evidence shows that helminth infection can improve the outcome of SARS-CoV-2 infection, and this phenotype is associated with type 2 microenvironment (11). Due to the ability of helminths (e.g., *Trichinella spiralis*, Ts) to effectively trigger an anti-inflammatory, immune response of type 2 that is mediated by type 2 cells and the cytokines they release, such as interleukin -4 (IL-4), IL-5, IL-9, and IL-13 (12), this mechanism might help alleviate circulatory compromise and lung damage.

In this study, we evaluated the effect of helminth Ts infection on TNF-α- and IFN-γ-mediated cytokine shock. Data showed that Ts-induced protection against TNF-α and IFN-γ shock was IL-9 dependent but IL-4Rα independent. We found that treatment of IL-9 protected against SARS-CoV-2 infection-related cytokine storm. Furthermore, Ts-derived products had better efficacy than IL-9 during SARS-CoV-2 infection. Our study highlights the value of this helminth as both a source of next-generation biologics and a tool for druggable target discovery.

## RESULTS

### Helminth induces protection against TNF-α and IFN-γ shock

**Fig 1:**
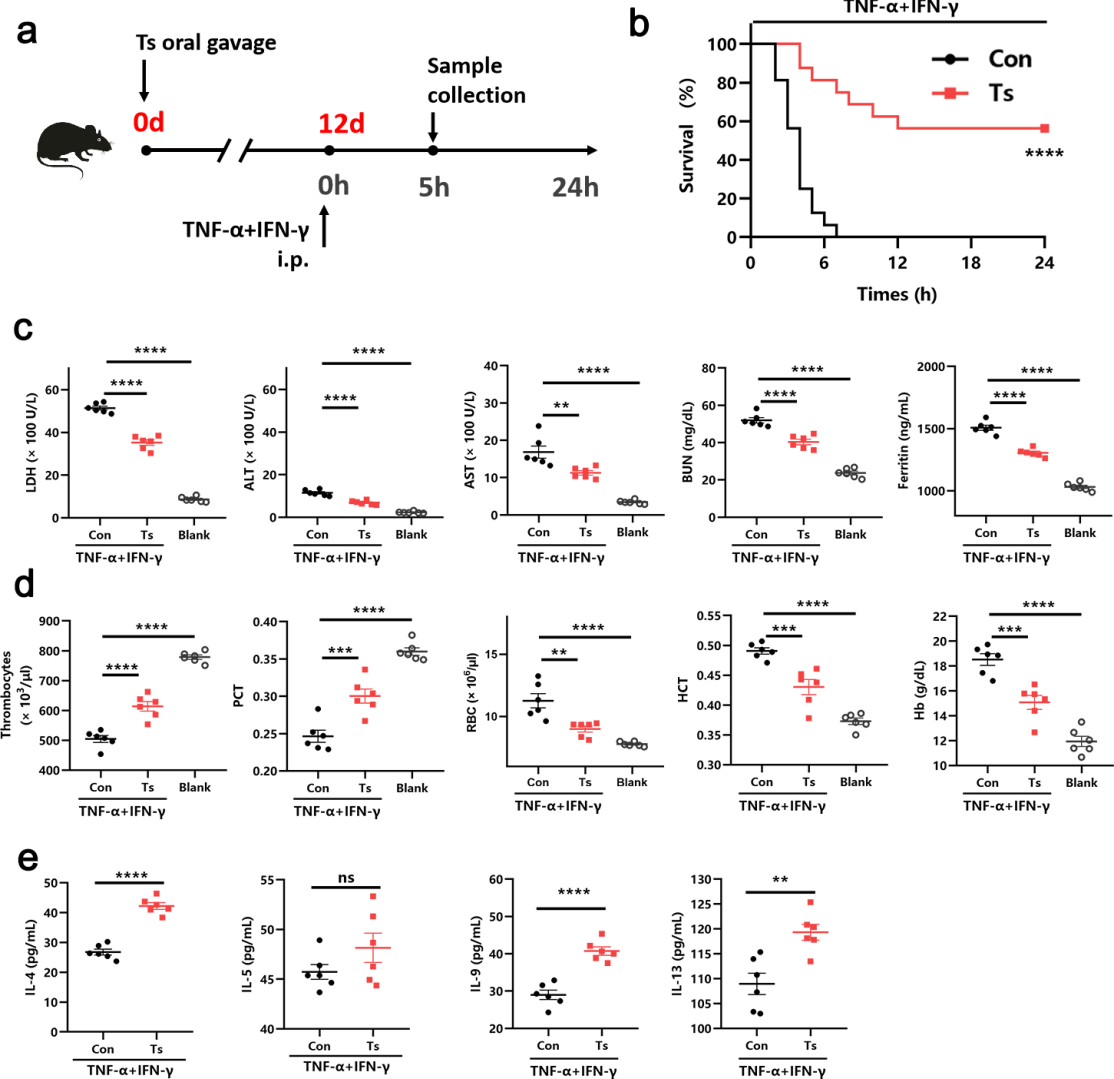
Helminth induces protection against TNF-α and IFN-γ shock. (**a**) Experimental design to determine whether helminth Ts infection protected against TNF-α and IFN-γ shock. i.p., intraperitoneal. (**b**) Survival of Ts-infected 6- to 8-week-old mice after i.p. injection of TNF-α and IFN-γ (*n* = 16). (**c**) Analysis of serum levels of LDH, ALT, AST, BUN, and ferritin in mice injected with TNF-α and IFN-γ after 5 h (*n* = 6). (**d**) The number of thrombocytes, PCT, RBC count, HCT, and Hb concentration in the blood of mice injected with TNF-α and IFN-γ after 5 h (*n* = 6). (**e**) Serum levels of type 2 cytokines, including IL-4, IL-5, IL-9, and IL-13, in mice injected with TNF-α and IFN-γ after 5 h (*n* = 6). Data are representative of at least two independent experiments. **P < 0.01; ***P < 0.001; ****P < 0.0001. Analysis was performed using the survival curve comparison (log-rank [Mantel-Cox] test) (**b**), the one-way analysis of variance (**c and d**), or the *t*-test (**e**). Data are shown as mean ± SEM (b–e). ns, no significance.

To evaluate the effect of helminth against cytokine storm, we developed a model of cytokine storm by the combination of TNF-α and IFN-γ (8) 12 d after helminth Ts infection (Fig. 1a). While injecting with TNF-α and IFN-γ led to 100% mortality, we observed a significantly improved survival in Ts-infected mice (Fig. 1b). Levels of serum lactate dehydrogenase (LDH), alanine aminotransferase (ALT), aspartate aminotransferase (AST), blood urea nitrogen (BUN), and ferritin were elevated until death in patients who succumbed to COVID-19 (13, 14). Similarly, we observed increased LDH, ALT, AST, BUN, and ferritin in the serum of the control mice injected with TNF-α and IFN-γ compared with the blank group, whereas Ts infection significantly reduced the levels of all laboratory parameters elevated by TNF-α and IFN-γ (Fig. 1c). Thrombocytopenia is associated with an increased risk of severity and mortality of COVID-19 (15). The complete blood counts revealed an increase in the number of thrombocytes and percentage of plateletcrit (PCT) in the blood of mice infected with Ts compared with controls (Fig. 1d). We also observed that Ts-infected mice had fewer red blood cell (RBC) count, hematocrit (HCT) percentage, and hemoglobin (Hb) levels (Fig. 1d). It is worth noting that Ts infection alone did not statistically change the above laboratory parameters levels (data not shown). These results suggested that Ts improves the outcome of TNF-α- and IFN-γ-induced severe disease. To confirm whether type 2 cytokines IL-4, IL-5, IL-9, and IL-13 contributed to helminth-induced protection against TNF-α and IFN-γ shock, we compared the levels of cytokines in the presence or absence of the helminth infection. Compared with the controls, Ts significantly increased the levels of IL-4, IL-9, and IL-13, but not IL-5 in circulation (Fig. 1e), indicating that IL-4Rα (a common subunit receptor for IL-4 and IL-13) and IL-9 might be involved in helminth-induced protection.

### Ts-induced protection against TNF-α and IFN-γ shock was IL-9 dependent but IL-4Rα independent

**Fig 2:**
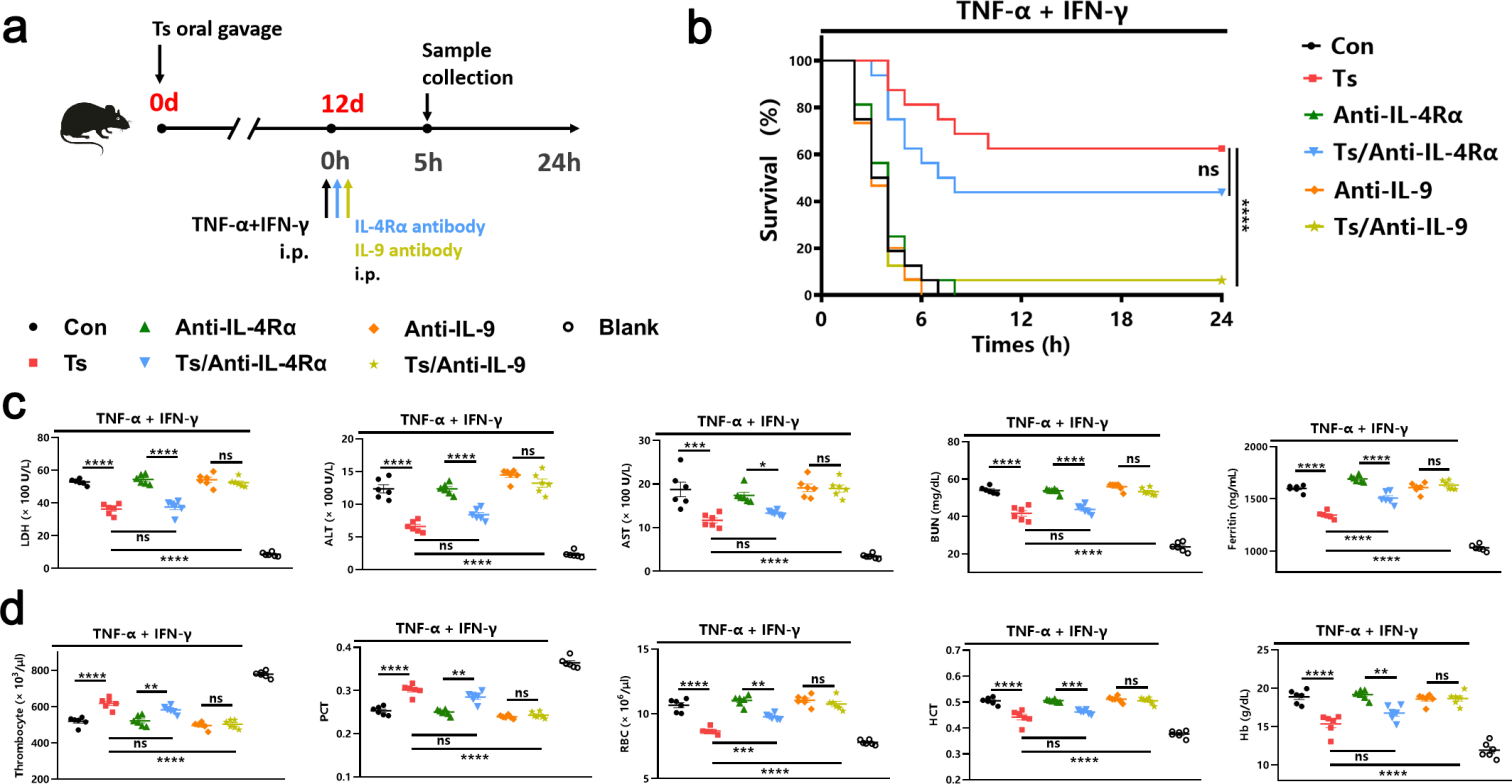
Helminth Ts-induced protection against TNF-α and IFN-γ shock was IL-9 dependent but IL-4Rα independent. (**a**) Experimental design to investigate the role of IL-4Rα and IL-9 in Ts-induced protection against TNF-α and IFN-γ shock. i.p., intraperitoneal injection. (**b**) Survival of Ts-infected mice with IL-4Rα or IL-9 blockage after injection of TNF-α and IFN-γ (*n* = 16). (**c**) Analysis of serum levels of LDH, ALT, AST, and BUN in mice injected with TNF-α and IFN-γ after 5 h (*n* = 6). (**d**) The number of thrombocytes, PCT, RBC count, HCT, and Hb concentration in the blood of mice injected with TNF-α and IFN-γ after 5 h (*n* = 6). Data are representative of at least two independent experiments. ns, no statistical significance, *P < 0.05; **P < 0.01; ***P < 0.001; ****P < 0.0001. Analysis was performed using the survival curve comparison (log-rank [Mantel-Cox] test) (**b**) or the one-way analysis of variance (**c and d**). Data are shown as mean ± SEM.

Next, we sought to confirm this hypothesis through the use of neutralizing antibodies (Fig. 2a). As shown in Fig. 2b, mice with blocking of IL-4Rα were still resistant to mortality induced by the cytokine shock, indicating helminth elicits protection independently of IL-4Rα. However, mice with IL-9 blockage only exhibited 1/16 survival during cytokine shock (Fig. 2b). All laboratory parameters induced by TNF-α and IFN-γ were significantly rescued in mice treated with IL-4Rα antibody (Fig. 2c and d), indicating helminth-elicited protection can occur independently of IL-4Rα. However, no significant differences were observed between Ts-infected mice with or without IL-9 blockage (Fig. 2c and d). These results suggest that Ts-mediated protection was IL-9 dependent but IL-4Rα independent.

### Recombinant IL-9 (rIL-9) treatment improved the outcome of TNF-α and IFN-γ shock

**Fig 3:**
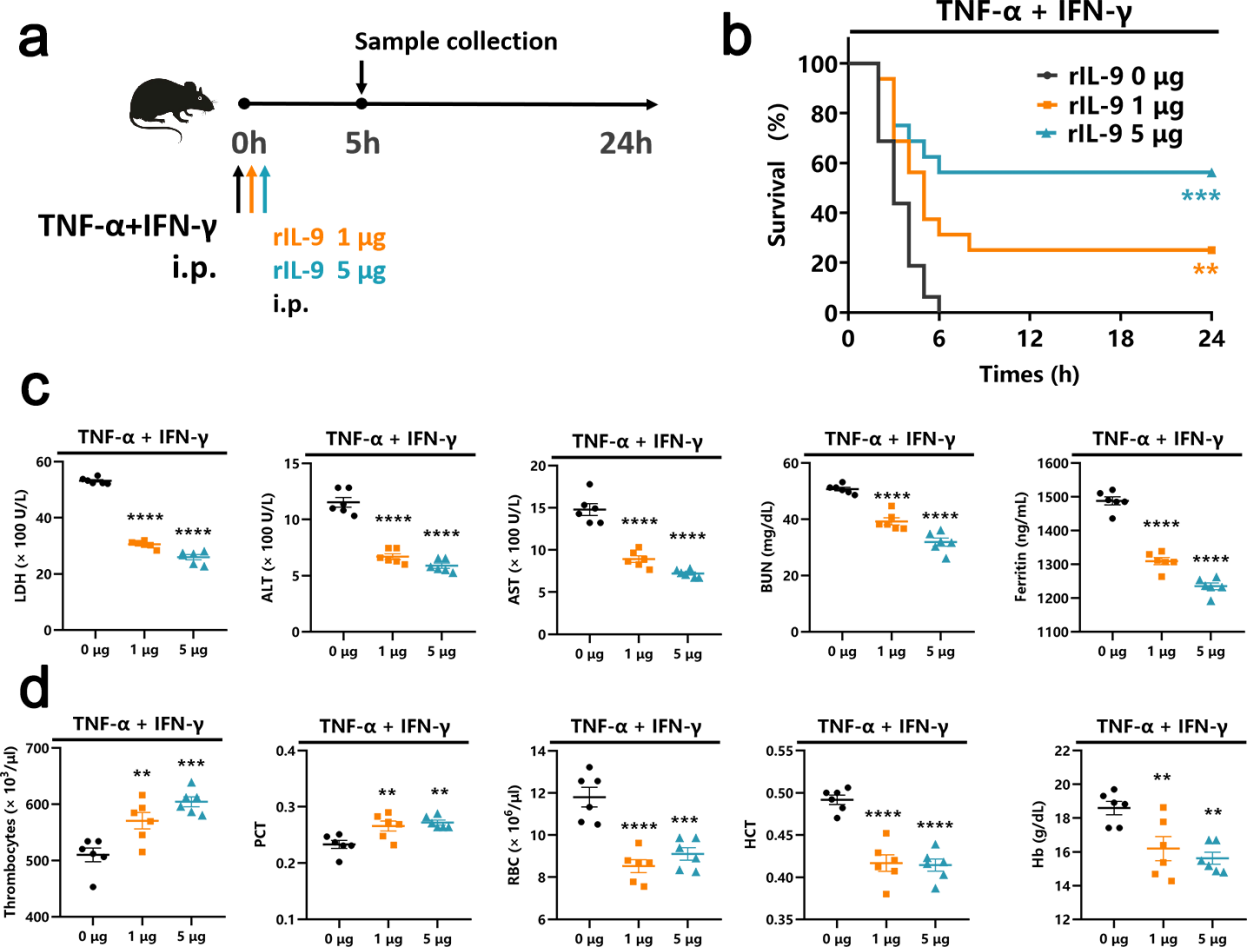
rIL-9 treatment improved the outcome of TNF-α and IFN-γ shock. (**a**) Experimental design to determine whether rIL-9 protected against TNF-α and IFN-γ shock. (**b**) Survival of rIL-9-treated mice after intraperitoneal (i.p.) injection of TNF-α and IFN-γ (*n* = 16). (**c**) Analysis of serum levels of LDH, ALT, AST, and BUN in mice injected with TNF-α and IFN-γ after 5 h (*n* = 6). (**d**) The number of thrombocytes, PCT, RBC count, HCT, and Hb concentration in the blood of mice injected with TNF-α and IFN-γ after 5 h (*n* = 6). Data are representative of at least two independent experiments. ns, no statistical significance, **P < 0.01; ***P < 0.001; ****P < 0.0001. Analysis was performed using the survival curve comparison (log-rank [Mantel-Cox] test) (**b**) or the one-way analysis of variance (**c and d**). Data are shown as mean ± SEM.

In the context of TNF-α and IFN-γ shock, the C57BL/6 mice were administered with different doses (1 µg or 5 µg per mouse) of rIL-9 via an intraperitoneal route (Fig. 3a). Data showed that treatment of rIL-9 significantly improved survival in a dose-dependent manner (Fig. 3b). We also observed dose-dependent effects of rIL-9 on the levels of LDH, ALT, AST, BUN, and ferritin in the serum (Fig. 3c). The complete blood counts revealed an increase in the number of thrombocytes and percentage of PCT in the blood of mice co-treated with IL-9, TNF-α, and IFN-γ compared with the TNF-α- and IFN-γ-treated group. Treatment with rIL-9 significantly reduced the levels of RBC, HCT, and Hb (Fig. 3d). Taken together, rIL-9 has a beneficial effect on TNF-α and IFN-γ shock.

### Both rIL-9 and Ts excretory/secretory (TsES) products could protect against SARS-CoV-2 infection in K18-hACE2 mice

**Fig 4:**
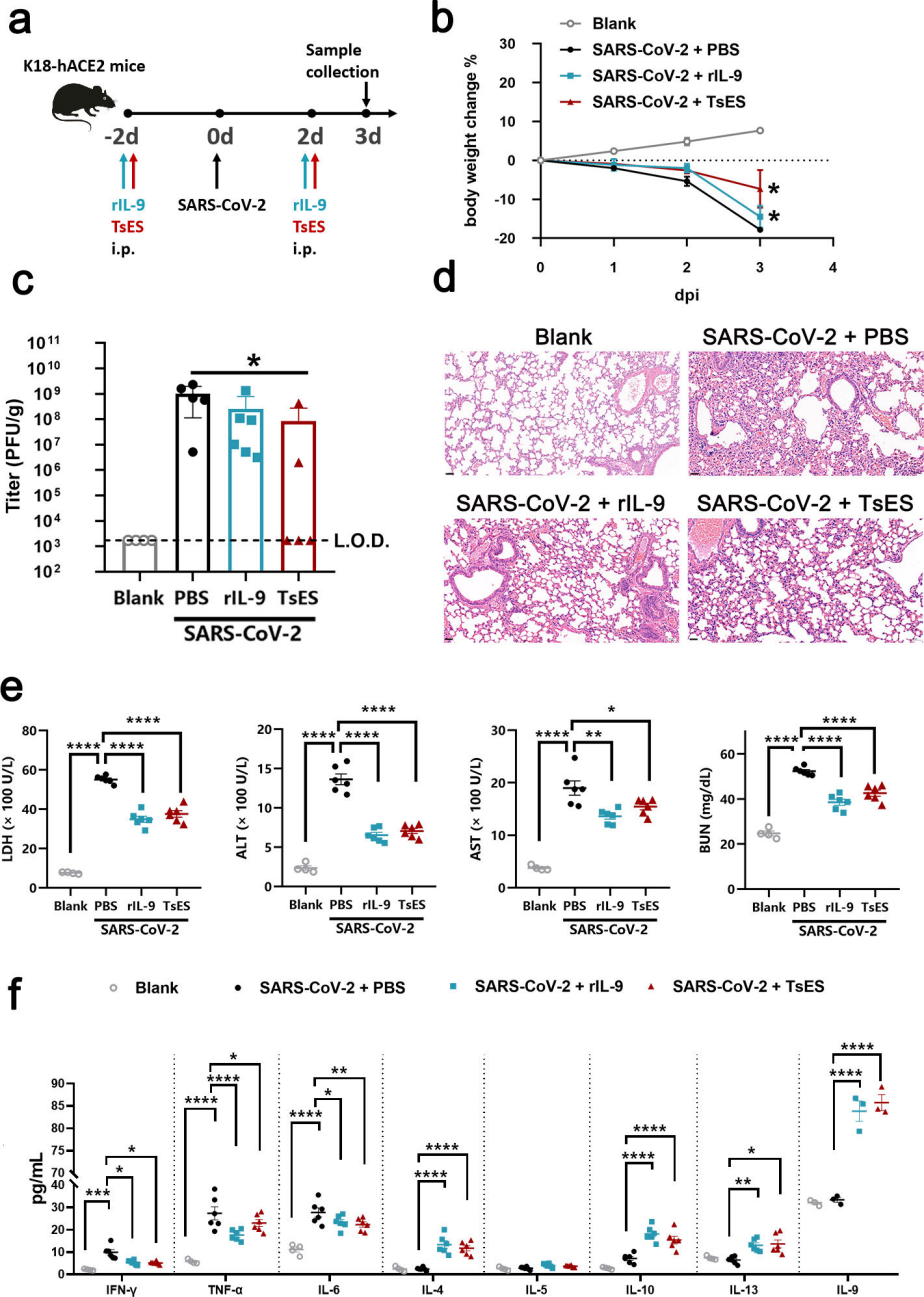
Both rIL-9 and helminth TsES products could protect against SARS-CoV-2 infection in K18-hACE2 mice. (**a**) Experimental design to determine whether rIL-9 or TsES protected against SARS-CoV-2 infection in 5- to 6-week-old K18-hACE2 mice. (**b**) Percent changes of body weights of rIL-9- or TsES-treated K18-hACE2 mice after SARS-CoV-2 infection (*n* = 4–6). Dashed lines represent the limit of detection. (**c**) Lung viral load of mice after SARS-CoV-2 infection (*n* = 4–6). (**d**) Representative micrographs of histological sections of lungs. Scale bars, 50 µm. (**e**) Analysis of serum levels of LDH, ALT, AST, and BUN of mice infected with SARS-CoV-2 after 3 d (*n* = 6). (**f**) Analysis of serum levels of cytokines of mice infected with SARS-CoV-2 after 3 d (*n* = 3–6). ns, no statistical significance, *P < 0.05; **P < 0.01; ***P < 0.001; ****P < 0.0001. Analysis was performed using a test (**b**) or the one-way analysis of variance (**c, e, and f**). Data are shown as mean ± SEM.

We further evaluated whether rIL-9 or TsES products protected SARS-CoV-2 infection-related cytokine storm in K18-hACE2 mice (Fig. 4a). Mice treated with rIL-9 or TsES displayed less body weight loss. Moreover, TsES had better efficacy than rIL-9 (Fig. 4b). Compared to placebo, both rIL-9 and TsES reduced the viral load in the lung, especially in the TsES-treated group; the viral load in the lung was undetectable (under limit of detection [L.O.D.]) in 60% (3/5) of infected mice (Fig. 4c). And mice infected with SARS-CoV-2 developed pathological changes in the lungs, such as pulmonary hemorrhage, widening of alveolar septa, and varied degrees of lymphocyte infiltration. The improvements were observed in the histopathology in the lungs of the mice treated with rIL-9 or TsES (Fig. 4d). SARS-CoV-2 infection increased the levels of LDH, ALT, AST, and BUN. These parameters were decreased by administration of rIL-9 or TsES (Fig. 4e). Both rIL-9 and TsES significantly decreased pro-inflammatory type 1 cytokines (IFN-γ, TNF-α, and IL-6) induced by SARS-CoV-2. The levels of type 2 cytokines (IL-4, IL-9, and IL-13), but not IL-5, were significantly elevated by rIL-9 or TsES. Compared with placebo treatment, the anti-inflammatory cytokine IL-10 production could be stimulated at a higher level by rIL-9 or TsES (Fig. 4f). These findings demonstrated that both rIL-9 and TsES application could ameliorate SARS-CoV-2 infection-related cytokine storm.

## DISCUSSION

Severe COVID-19 fatalities are associated with cytokine storms. Cytokine storm syndromes, such as those seen in COVID-19, are reflected in TNF-α and IFN-γ shock (8). Thus, we utilized this model to investigate the influence of helminth infection on COVID-19-related cytokine storms. We found that prior helminth Ts infection improved the outcome of TNF-α and IFN-γ shock, accompanied by an increase in type 2 cytokine levels (IL-4, IL-13, and IL-9). Unexpectedly, Ts-induced protection was independent of IL-4Rα (a common subunit receptor for IL-4 and IL-13). IL-9 was required for Ts-induced protection against TNF-α and IFN-γ shock. Treatment with IL-9 could protect against not only TNF-α and IFN-γ shock but also SARS-CoV-2 infection. Importantly, Ts-derived products had better efficacy than IL-9 treatment on SARS-CoV-2 infection. We conclude that these helminth-derived molecules warrant further development as therapeutics for the treatment of cytokine storm syndromes, including COVID-19.

IL-9 was first described in the late 1980s as a member of cytokines (16). IL-9 production was first associated with the T helper 2 (Th2) phenotype (17). IL-9 mediates anti-helminth immunity through the local or systemic production of IL-9. During *Trichuris muris* and *Nippostrongylus brasiliensis* infections, IL-9 is required for worm expulsion (18, 19). IL-9 facilitates helminth Ts expulsion during the enteric phase of infection (20). More evidence suggests that Th9 cells are a specialized subset of T cells dedicated to producing IL-9 (21). In the intestines, Ts infection induces type 2 innate lymphoid cells (ILC2) (22), which is also the source of IL-9 (23, 24). Type 1 and 2 immune responses are antagonistic. It is interesting to note that IL-9 attenuates disease development in a Th1 cell-mediated inflammatory model (25). IL-9 also protects mice from bacterial cytokine shock, and this effect is correlated with the down-regulation of TNF-α and IFN-γ (26). Depletion of IL-9 at challenge tends to enhance TNF-α and IFN-γ production in the lungs of mice infected with respiratory syncytial virus, suggesting that IL-9 is critical for inhibiting exacerbating pro-inflammatory cytokines (27).

During SARS-CoV-2 infection, patients exhibit systemic symptoms of varying severity associated with the release of numerous pro-inflammatory cytokines (13). Stimulation of peripheral blood mononuclear cells from COVID-19 patients with helminth antigens is associated with reduced IFN-γ and TNF-α production and higher IL-10 levels (28). These findings are consistent with our study that IL-9 protects against COVID-19-related cytokine storms. Our work demonstrated that pre-treatment of IL-9 could reduce type 1 cytokines levels through elevated type 2 cytokines and IL-10 levels in K18-hACE2 mice infected with SARS-CoV-2. Intestinal helminth *Heligmosomoides polygyrus bakeri* infection can also induce systemic expression of IL-10, which impairs IFN-γ^+^ T cell responses induced by SARS-CoV-2 vaccine (29). Notably, IL-10 dependence on IL-9 production has also been described (30). Future investigations on understanding the roles of a combination of IL-10 and IL-9 in contributing to protection against cytokine storm may provide novel insights for better approaches to treat severe SARS-CoV-2 infection.

In addition, helminth parasites could modulate the immune reactivity to SARS-CoV-2 peptides by maintaining SARS-CoV-2-reactive CD8^+^ cytotoxic T cells (28). ILC2-derived IL-9 could activate CD8^+^ T cells (31). In contrast, the deletion of IL-4Rα expands CD8^+^ T cells (32), which may be a reason why IL-4Rα can only play a partial protective role in COVID-19-related cytokine storms. It will be of great interest to examine whether Ts infection-induced IL-9-producing ILC2s play a critical role in controlling SARS-CoV-2 infection by maintaining CD8^+^ T cells. However, recent studies reveal a pathologic role of IL-9 in SARS-CoV-2 infection (33). They showed that IL-9 treatment aggravates SARS-CoV-2-associated airway inflammation through the intranasal route for 24 h before euthanizing the mice. Indeed, IL-9 is an important mediator in asthma (34), especially when administered intranasally. Interestingly, people with asthma are at lower risk of being infected with COVID-19 compared to those without asthma (35). In contrast, we administered by intraperitoneal injection of IL-9. Because the intraperitoneal route is regarded as a systemic route (36), it seems to fight systemic inflammatory response syndrome. IL-9 was pre-treated 48 h before SARS-CoV-2 infection to remodel the type 2 environment in the host. Consistent with previous studies (11), a preexisting type 2 environment is beneficial rather than detrimental to subsequent SARS-CoV-2 infection.

In addition, mounting evidence suggests that *Trichinella* spp. can effectively regulate immune responses targeted at the parasite, creating an environment that reduces inflammation and supports balance (37, 38). Ts infection could ameliorate influenza virus-induced inflammation in the lungs (39) and *Pseudomonas aeruginosa*-induced pneumonia through a Th2-type response. Although we have not directly shown the outcome of coinfection of Ts and SARS-CoV-2, we directly observed the protective efficacy of TsES, and this phenotype correlated with lower type 1 cytokines and higher type 2 cytokines. Through the secretion of ES products by muscle larvae, this parasite establishes enduring communication with its host. These products play a crucial role in interacting with immune cells, ultimately driving the processes of parasitism and modulation of the immune response (40). The molecules present in ES actively stimulate the Th2 pathway and anti-inflammatory responses (41). Since treatment with live helminths carries certain risks, identification of immunomodulatory molecules of TsES is important using genomic, proteomic, and molecular methods, as they serve as potential therapeutic drugs for anti-inflammation. For example, a recombinant 53 kDa glycoprotein from TsES (rTsP53) displays anti-inflammatory characteristics and protects mice from endotoxemia induced by lipopolysaccharides (LPS) by reducing levels of pro-inflammatory agents (TNF-α, IL-1β, and IL-6) (42). In a model of Th1-mediated intestinal inflammation induced by 2,4,6-trinitrobenzene sulfonic acid (43), rTsP53 reduces the levels of IFN-γ and TNF-α in sera of treated mice (44). RTsP53 treatment could alleviate LPS-induced acute lung injury, down-regulated pro-inflammatory mediators (TNF-α, IL-1β, and IL-6), and up-regulated anti-inflammatory mediators (IL-4, IL-10, and IL-13) (45). Future efforts to explore whether this protein has a therapeutic effect on COVID-19-related cytokine shock syndromes are needed.

**Fig 5:**
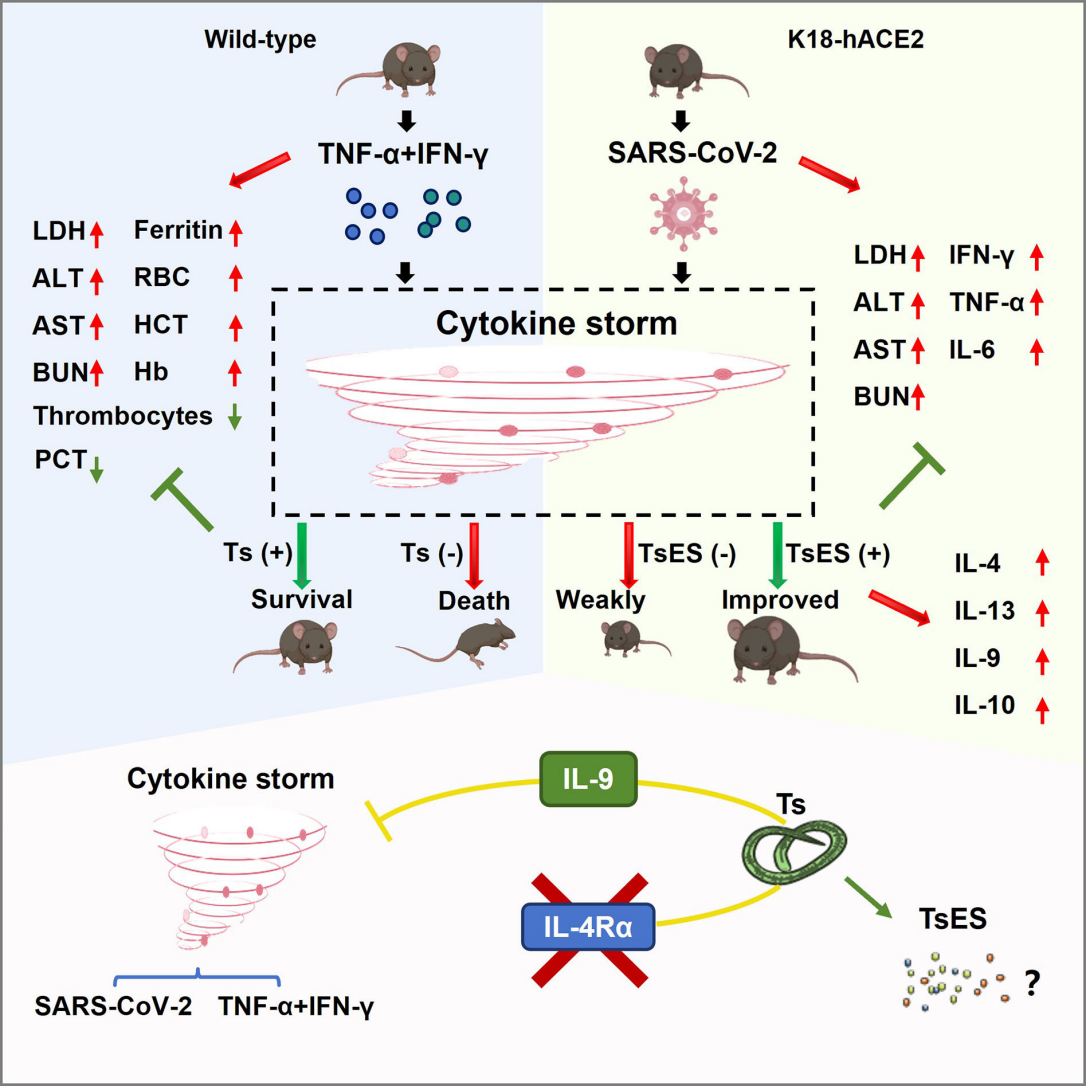
Helminth induces protection against COVID-19-related cytokine storms in an IL-9-dependent way. In the left panel, the treatment of TNF-α and IFN-γ remodeled cytokine storm in wild-type mice. The administration with Ts led to the survival of the mice suffering from cytokine storm, with an improved effect on all laboratory parameters. In the right panel, SARS-CoV-2 infection resulted in the death of K18-hACE2 mice, while the administration with TsES alleviated the adverse inflammatory response of SARS-CoV-2 infection. As a result, the symptom of infected K18-hACE2 mice was improved. The bottom summarizes the function of Ts/TsES-induced IL-9 in cytokine storm improvement. Nonetheless, which components function in a therapeutic role is still unknown. Future efforts should focus on the identification of the therapeutic components of helminths.

In summary, our data emphasize the significance of IL-9 in protecting from cytokine storm syndromes associated with SARS-CoV-2 infection (Fig. 5). Furthermore, further studies that identify these anti-inflammatory pathways and molecules induced by helminths could offer new agents to alleviate adverse pathological inflammation related to infections, including COVID-19, as helminths can also benefit their hosts through immunoregulatory networks that resolve inflammation.

## MATERIALS AND METHODS

### Mice

Six- to 8-week-old C57BL/6 mice were purchased from the Norman Bethune University of Medical Science. Five- to 6-week-old K18-hACE2 mice were obtained from GemPharmatech Co., Ltd.

### Helminth infection

Methods for maintenance, recovery, and infection of Ts were previously described (46). Ts were checked by microscope for motility, and their numbers were quantified before use. Mice were gavaged with 200 Ts or phosphate buffered saline (PBS) (control) to establish the models of helminth infection. Ts muscle larvae were washed and incubated separately in prewarmed serum-free RPMI 1640 medium containing 2 mM L-glutamine, 100 U/mL penicillin, and 100 mg/mL streptomycin at 37°C under 5% atmospheric CO_2_ for 24 h. After centrifugation, the supernatant containing TsES products was dialyzed and concentrated.

### *In vivo* TNF-α and IFN-γ-induced shock

Twelve days after Ts infection, mice were injected intraperitoneally with TNF-α (10 µg, PeproTech, # 315-01A) and IFN-γ (20 µg, PeproTech, # 315-05) diluted in Dulbecco’s phosphate buffered saline (DPBS) as described (8). Anti-mouse IL-9 monoclonal neutralizing antibody (100 µg, BioXCell, #MM9C1), anti-mouse IL-4Rα monoclonal neutralizing antibody (100 µg, BD Biosciences, # 552288), or isotype antibodies were injected intraperitoneally with TNF-α and IFN-γ. Animals were under permanent observation, and survival was assessed every 30 min. Blood was collected 5 h after cytokine injection. Blood composition was analyzed using an automated hematology analyzer. Serum LDH, AST, ALT, BUN, and ferritin were analyzed by colorimetry using respective kits (LDH, # A11A01824; ALT, # A11A01627; AST, # A11A01629; and BUN, # A11A01641, all from HORIBA; and ferritin, Abcam, # ab157713) according to the manufacturer’s instructions. Type 2 cytokine IL-4 (R&D Systems, # SM4000B), IL-5 (R&D Systems, # SM5000), IL-9 (Biolegend, #434806), and IL-13 (R&D Systems, #SM1300CB) were measured by enzyme linked immunosorbent assay (ELISA) kits according to the manufacturer’s instructions.

### rIL-9 treatment in TNF-α and IFN-γ–induced shock

C57BL/6 mice were administered intraperitoneally 0, 1, or 5 µg of murine rIL-9 (R&D Systems, # 409 ML-050) together with TNF-α and IFN-γ injection. Animals were under permanent observation, and survival was assessed every 30 min. Blood was collected 5 h after cytokine injection. Blood composition was analyzed using an automated hematology analyzer. Serum LDH, ALT, AST, and BUN were analyzed by colorimetry using respective kits according to the manufacturer’s instructions (8).

### Animal infection and treatment with rIL-9 or TsES

Animal infection was performed in the animal biosafety level 3 facility at the Wuhan Institute of Virology. K18-hACE2 mice (5–6 weeks old) were intranasally infected with 5.0 × 10^4^ plaque-forming units (PFU) of SARS-CoV-2 WIV04 strain (IVCAS 6.7512) (PMID: 32015507). The mice were intraperitoneally administered with rIL-9 or TsES at 2 days before infection and 2 days post-infection (d.p.i.), respectively. The body weight was measured daily, and the lungs, nasal turbinates, and tracheas were harvested to determine the viral loads at 3 d.p.i. Lung tissues were fixed with 10% neutral formaldehyde for hematoxylin and eosin staining. Cytokines (IFN-γ, TNF-α, IL-4, IL-5, IL-6, IL-10, and IL-13) were assessed in serum using a ProcartaPlex Luminex kit (R&D Systems, # LXR-LXSAMSM-07) according to the manufacturer’s instructions and measured using a MagPix Instrument. Quantitation of IL-9 in serum was measured by ELISA kits (Biolegend, #434806) according to the manufacturer’s instructions.

### Titration

Viral titers were determined by plaque assay on Vero E6 cells as previously described (47). Briefly, the samples were serially 10-fold diluted using Dulbecco’s modified Eagle’s medium containing 2.5% fetal bovine serum (FBS) plus 2% penicillin/streptomycin, and 100 µL of each dilution was added to Vero E6 cells in 24-well plates. After 1-h incubation of the plates, the inoculums were replaced by fresh methylcellulose overlay containing 2% FBS. The plates were incubated at 37℃ for 3 d, followed by fixation with 8% paraformaldehyde. Then, the plates were stained with 1% crystal violet, and visible plaques were counted to determine viral titers as PFU.

### Statistical analysis

All statistical tests were performed as described in the indicated figure legends using Prism 8.0. Analysis was performed using the survival curve comparison (log-rank [Mantel-Cox] test), the *t*-test, or the one-way analysis of variance. Data are shown as mean ± SEM.
